# Reliability of 30-s Chair Stand Test with and without Cognitive Task in People with Type-2 Diabetes Mellitus

**DOI:** 10.3390/ijerph17041450

**Published:** 2020-02-24

**Authors:** Sabina Barrios-Fernández, Jorge Pérez-Gómez, María del Carmen Galán-Arroyo, Jairo Señorán-Rivera, Rubén Martín-Carmona, María Mendoza-Muñoz, Miguel Ángel García-Gordillo, Francisco Javier Domínguez-Muñoz, José Carmelo Adsuar

**Affiliations:** 1 Faculty of Nursing and Occupational Therapy, University of Extremadura, 10003 Cáceres, Spain; 2 Faculty of Sport Sciences, University of Extremadura, 10003 Cáceres, Spain; mamendozam@unex.es (M.M.-M.); fjdominguez@unex.es (F.J.D.-M.); 3 Exercise Looks after You Program (ELAY), 10003 Cáceres, Spain; mamengalan.tq@gmail.com (M.d.C.G.-A.); jairoseriv@gmail.com (J.S.-R.); mcruben@hotmail.com (R.M.-C.); 4 Facultad de Administración y Negocios, Universidad Autónoma de Chile, sede Talca 3467987, Chile; miguel.garcia@uautonoma.cl

**Keywords:** intraclass correlation coefficient, standard error of measurement, physical function, dual-task

## Abstract

Background: Reliability refers to the precision of an assessment, so it is a critical topic to take the right decisions related to health management. People usually perform several tasks at the same time in their daily life. The aim of this study was to examine the reliability of the 30-s chair stand test in people with type 2 Diabetes Mellitus (T2DM) with test–retest, with and without dual-task (motor + cognitive task). Methods: Twenty-six subjects with T2DM and 30 subjects without T2DM performed the 30-s Chair Stand Test (30sCST) in which they must sit and stand as many times as possible in 30 s. They performed the test in the usual way (30sCST) and also with an additional cognitive task (30sCST-DT). A retest was conducted 7–14 days later. Results: Relative reliability was excellent in both groups (intraclass correlation coefficient > 0.9). In 30sCST-DT, relative reliability was high in the T2DM group (intraclass correlation coefficient > 0.7) and excellent in subjects without T2DM (intraclass correlation coefficient > 0.9). Conclusions: The 30sCST and the 30sCST-DT tests are reliable tools for people with T2DM to measure changes after an intervention. The smallest real difference was 15% and 20% upper in the T2DM group in the 30sCST and 30sCST-DT tests, respectively.

## 1. Introduction

Non-insulin-dependent Type 2 Diabetes Mellitus (T2DM) is a controllable chronic disease characterized by a chronic hyperglycemia that occurs when the body cannot use or produce insulin properly. Its symptoms include thirst, polyuria, blurred vision, and weight loss [1]. Nowadays, essential advances are happening both in diagnosis and management [2] because of the high prevalence worldwide [3], upward trend [4], and high sanitary costs [5]. For proper T2DM management, adequate glycemic control, cardiovascular risk factors management, early treatment of complications and related comorbidities are needed [6]. T2DM complications include heart and blood vessel disease, neuropathy, kidney disease, skin and eye damage, sleep apnea, bone metabolism impairments, mood disorders, and cognitive impairment [7]. Thus, maintaining a healthy lifestyle with weight control, balanced diet, quitting smoking, and physical activity are essential [8].

Physical activity improves glycemic control and reduces cardiovascular diseases and mortality [9]. Despite this, people with T2DM usually have poor physical condition, and they do not practice enough exercise [9,10,11]. In addition to physical benefits, psychological benefits are also important, improving depressive symptomatology [12,13]. Thus, health professionals need tests to adequately measure physical condition and to prescribe individualized exercise programs [10], but few studies have evaluated the reliability of fitness tools in the T2DM population [14]. One physical test is the 30-s chair stand test (30sCST) which consists of getting up and sitting down from a chair as many times as possible in 30 s [15]. It is widely used to measure strength and endurance in lower limbs, discriminating between functional states, and providing information about fatigue [16,17,18].

Having a T2DM diagnosis influences cognitive function: accelerates the aging process, increases the risk of dementia, and reduces processing speed, learning and memory [19,20,21,22]. In addition, functionality in daily life activities can be affected [19,23]. All this leads to the dual-task (DT) paradigm defined as performing several activities simultaneously [24]. DT can be motor–cognitive, cognitive–cognitive or motor–motor [25]. In their daily lives, people usually divide their attention between performing motor tasks and cognitive activity at the same time [26,27,28], so it is necessary to determine the psychometric properties of the tests, including the reliability. Therefore, the main objective was to evaluate the test–retest reliability of performing the 30sCST test in people with T2DM, with and without DT (30sCST-DT).

## 2. Materials and Methods

### 2.1. Participants

Participants were recruited through the public health “The Exercise Looks After You” Program (ELAY) [29]. Inclusion criteria to participate were having a T2DM diagnosis, not having functional difficulties in walking, being participants in the ELAY Program, and providing the written informed consent. A total of 36 subjects with T2DM, 11 men and 15 women, aged 62–82 years old, were included. Average Body Mass Index (BMI) was into the overweight or obesity range [30,31]. The other 30 participants without T2DM were selected as controls, including 15 men and 15 women (Table 1).

The sample size was calculated to achieve a power of 90 for an intraclass correlation coefficient (ICC) under the following assumptions: alpha = 0.05; the null hypothesis was that the ICC was good according to the criteria used (0.70) [32]. The alternative hypothesis was that the ICC was excellent (0.92) according to previous studies of healthy participants [15]. A minimum of 14 participants was required for each test session.

Protocols followed the Declaration of Helsinki updates [33], and the study was approved by the Committee on Biomedical Ethics of the University of Extremadura (106/2018).

### 2.2. Procedures

Sociodemographic and health data were collected. The 30sCST [15] was administered in the facilities where the ELAY Program runs. Participants were allowed to practice a trial before running the tests. The order to first perform the 30sCST or the 30sCST-DT was randomly determined. The cognitive task in the DT test consisted of performing 3-in-3 subtractions backwards, starting at number 100. The time required to complete the task was assessed using Chronojump (Chronojump-BoscoSystem^TM^). This system consists of free software that uses open hardware Chronopic [34,35]. Seven to fourteen days later, another measurement under the same conditions was taken (retest). Trained technicians evaluated the participants, keeping the same technician paired with the same participant in both measures.

### 2.3. Statistical Analysis

NCSSTM^TM^ Pass v.11 software (NCSS, LLC. Kaysville, UT, USA) was used to calculate the sample size. Microsoft Office^TM^ Excel v.16 (Microsoft Corporation, Redmond, Washington, DC, USA) and IBM^TM^ SPSS v.25 (International Business Machines Corporation, Armonk, New York, NY, USA) were used for data analysis. First, the Kolmogorov–Smirnoff and Shapiro–Wilks test were used to assess normality, and a normal distribution was considered with *p*-value > 0.05. All the variants followed a normal distribution (Table 2). A paired-samples *t*-test was used to examine the differences between both values test and retest. Relative reliability was calculated using the ICC with a 95% confidence interval across the two sessions. ICC interpretation was made with Munro’s criteria: 0.50–0.69 moderate, 0.70–0.89 high, and <0.90, excellent [32]. Absolute reliability was determined with the standard error of measurement (SEM) and the smallest real difference (SRD) scores at 95% confidence interval (SRD_95_) with the following equations [36]:

(1) $$\mathrm{SEM}\;=\;\mathrm{SD}\;\sqrt{(1-\mathrm{ICC}})$$

In this equation, the standard deviation (SD) is the mean SD of the test and the retest, and ICC is the reliability coefficient. SRD_95_ = 1.96 √2SEM. The 1.96 in the SRD_95_ equation represents the z-score at the 95% confidence level. Both SEM and SRD are indices that express reliability in absolute terms (with the same unit of measurement). Although relative reliability indices have been widely used, absolute indices show some advantages, such as the ease to extrapolate the results to other individuals and to compare reliability between different measurement tools. Both percentages were also calculated. Bland–Altman graphics were shown to assess systematic error [37].

## 3. Results

Table 2 shows mean and standard deviation (SD) of the repetitions performed by the participants in test–retest in both tests, 30sCST and 30sCST-DT. A paired-samples *t*-test is also shown. No statistically significant differences were found between the two testing days for all outcomes of the study (*p* < 0.05).

In T2DM participants, relative reliability values (total, men, and women) were considered excellent for the 30sCST (>0.9). In the 30sCST-ST, total and women values were considered high and poor for men with T2DM [32]. In non-T2DM participants, all the values were considered excellent (see Table 3).

**Figure 1:**
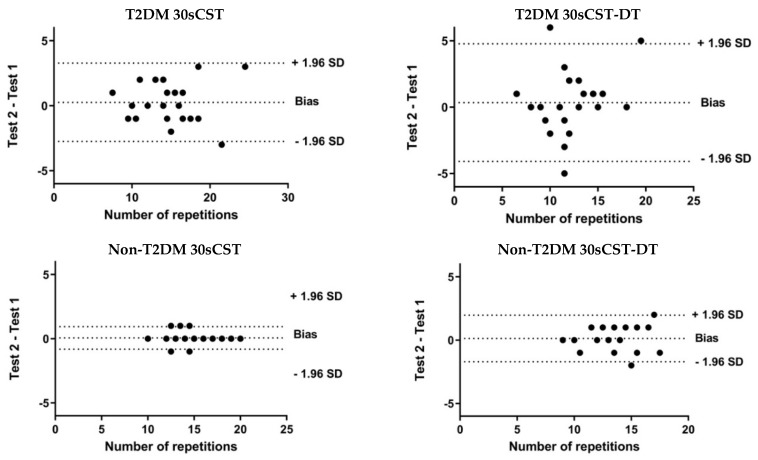
Bland–Altman’s graphs for 30-s chair stand test and 30-s chair stand test with and without dual-task.

The Bland–Altman’s graphs [37] illustrate the differences between test and retest measurements in T2DM and non-T2DM groups (Figure 1).

## 4. Discussion

The 30sCST is often used in the assessment of physical condition in adults due to its simplicity and good psychometric properties [38]. It is one of the recommended tests by the Centers for Disease Control for fall-risk screening [18]. In our study, we performed a test–retest to delimit the reliability of the 30sCST in the T2DM population compared with the non-T2DM population. One of the main findings was that the relative reliability in the 30sCST was considered excellent (ICC < 0.90) for both T2DM and non-T2DM populations. The first reliability data of the 30sCST were presented by their original authors [39] who referred an ICC = 0.84 for men (high) and ICC = 0.92 for women (excellent). Its reliability has also been tested with adults over 50 years, obtaining ICC = 0.66 (moderate) and SEM = 1.63 repetitions [40]. The 30sCST reliability has been checked in different specific populations. The reliability values in people with total hip arthroplasty [41] were ICC = 94 (excellent) and SEM = 0.4 repetitions. People diagnosed with dementia [42] scored ICC = 0.84 (high), SEM = 1.26, and SRD = 4.86 repetitions. Women with fibromyalgia [43] obtained high values (ICC = 0.87), SEM = 0.77 repetitions, and SRD = 2.14 repetitions. In population who suffered a stroke [44] it had excellent results (ICC = 0.91–0.97), SEM = 0.75, and SRD = 2 repetitions.

To our knowledge, only one study measured the reliability of the 30sCST in people with T2DM [14]. They also administered the hand grip strength test, the chair sit and reach test, the timed “up and go” test, the 6-min walk test and they found all of them were reliable with excellent values according to Munro [32]. Their results showed ICC = 0.92, SEM = 1.21, SRD = 3.35, while ours were ICC = 0.92, SEM = 1.08, SRD = 3, so we found similar values. The percentage of SEM is important for a correct interpretation of data because it indicates the degree of measurement noise. Our SEM value suggests that test–retest differences under 7.3% should be considered measurement noise and should not have clinical significance [45].

Regarding gender differences, %SEM is 5.9% for men and 7.9% for women. In the case of the 30sCST-DT, the SEM for the total sample was 12.8%, 15.7% for men and 10.4% for women. Regarding the %SRD, a general guideline for 30sCST in T2DM people should be that 20.1% of change should be indicative of genuine clinical change, while in the 30sCST-DT should be 35.4%.

Regarding DT, which is relevant because in our daily life we usually perform several tasks at the same time [46], we found that the number of repetitions in 30sCST-DT was lower than in 30sCST (total, men, and women). An explication for this is that participants put their attention into the cognitive task and consequently, the motor task became worst [47]. No studies were found about DT performance in the T2DM population. In our study, relative reliability for T2DM group was high (ICC = 0.73), showing better results in the women subgroup (ICC = 0.86) than in men (ICC = 0.4) [32]. All the values were excellent for the 30sCST-DT in non-T2DM (ICC < 0.9). Related to the DT topic and T2DM, one study which used a fitness test battery found preliminary evidence of poor balance, getting worse in DT and increasing the risk of falls [19,48].

Some limitations were found. Reliability can be affected by several factors, such as data processing and the variability associated with both technicians and participants [49]. To reduce it, technicians evaluated the same participant in both sessions, following a standardized protocol. The sample was one of convenience, so further studies should consider trying to include randomization and increasing the sample size with a larger number of T2DM men. We had no control over some environmental conditions, such as temperature during test sessions, and we could not take measurements at exactly the same time (morning or afternoon sessions were kept). Some key points for future studies should be trying to increase the time interval between the test–retest sessions to find out if reliability values are maintained. It also would be interesting to run the test in people with T2DM who suffer complications, such as diabetic neuropathy.

## 5. Conclusions

Both 30sCST and 30sCST-DT tests are reliable tools for people with T2DM to measure changes after an intervention. The SRD was 15% and 20% upper in the T2DM group in 30sCST and 30sCST-DT tests, respectively, compared to non-T2DM.

## Figures and Tables

**Table 1:** Characteristics of the participants included in the study.

	Total	Men	Women
*T2DM participants*			
Participant number	26	11	15
Age (years)	72.81 ± 5.96	74.92 ± 5.58	71.27 ± 5.94
Height (cm)	160.69 ± 8.1	166.74 ± 5.12	156.27 ± 7
Weight (kg)	74.02 ± 7.78	75.04 ± 8.69	73.27 ± 7.26
Body Max Index	28.75 ± 3.12	27.01 ± 3.02	30.03 ± 2.6
*Non-T2DM participants*			
Participant number	30	15	15
Age (years)	69.07 ± 5.5	70.80 ± 6.06	67.33 ± 4.35
Height (cm)	164.33 ± 7.23	168.04 ± 4.24	160.27 ± 7.41
Weight (kg)	74.47 ± 8.08	77.27 ± 7.09	71.67 ± 8.25
Body Max Index	27.62 ± 3	27.21 ± 1.84	28.03 ± 3.86

**Table 2:** Repetitions in the 30-s chair stand test and 30-s chair stand test with the dual-task test in two measurements (test–retest) taken 7–14 days later.

	Test Type	Test Mean ± ST (Repetitions)	Retest Mean ± ST (Repetitions)	*p*
*T2DM participants*				
Total	30sCST	14.77 ± 3.89	15.04 ± 3.76	0.381
	30sCST-DT	12.12 ± 2.76	12.46 ± 3.28	0.442
Men	30sCST	13.73 ± 2.61	13.91 ± 2.51	0.617
	30sCST-DT	11.73 ± 2.53	12.55 ± 2.38	0.341
Women	30sCST	15.53 ± 4.39	15.87 ± 4.36	0.485
	30sCST-DT	12.4 ± 2.98	12.4 ± 3.89	1
*Non-T2DM participants*				
Total	30sCST	15.23 ± 2.69	15.30 ± 2.65	0.423
	30sCST-DT	12.97 ± 2.34	13.1 ± 2.44	0.442
Men	30sCST	16.07 ± 3.11	16 ± 3.19	0.334
	30sCST-DT	13.53 ± 2.59	13.87 ± 2.56	0.207
Women	30sCST	14.4 ± 1.96	14.6 ± 1.85	0.189
	30sCST-DT	12.4 ± 1.99	12.33 ± 2.13	0.774

**Table 3:** Repetitions in the 30-s chair stand test and 30-s chair stand test with the dual-task test in the two measurements (test–retest) taken 7–14 days later.

	Test Type	ICC (95% CI)	SEM (Repetitions)	%SEM	SRD (Repetitions)	%SRD
*T2DM participants*						
Total	30sCST	0.92	1.08	7.3	3	20.1
	30sCST-DT	0.73	1.57	12.8	4.35	35.4
Men	30sCST	0.9	0.81	5.9	2.24	16.2
	30sCST-DT	0.4	1.90	15.7	5.27	43.4
Women	30sCST	0.92	1.24	7.9	3.43	21.8
	30sCST-DT	0.86	1.29	10.4	3.56	28.7
*Non-T2DM participants*						
Total	30sCST	0.99	0.27	1.7	0.74	4.8
	30sCST-DT	0.92	0.68	5.2	1.87	14.4
Men	30sCST	0.99	0.32	2	0.87	5.4
	30sCST-DT	0.93	0.68	5	1.89	13.8
Women	30sCST	0.95	0.43	2.9	1.18	8.1
	30sCST-DT	0.91	0.62	5	1.71	13.9

ICC: intraclass correlation coefficient; CI: confidence interval; SEM: standard error of measurement; SRD: smallest real difference.
